# Integrating Invasive and Noninvasive Imaging for Coronary Atherosclerosis: A Systematic Review With Pragmatic Algorithms From Anatomy to Physiology

**DOI:** 10.7759/cureus.112199

**Published:** 2026-07-07

**Authors:** Maurice Tiotsop, Douni O Roger, Utsab Panta, Joshua K Salabei

**Affiliations:** 1 Medicine, University of Louisville School of Medicine, Louisville, USA; 2 Internal Medicine, SparrowHospital/Michigan State University, Lansing, USA; 3 Cardiology, Baptist Health Hardin, Elizabethtown, USA

**Keywords:** coronary angiography, coronary atherosclerosis, coronary computed tomography angiography, fractional flow reserve, intravascular ultrasound, optical coherence tomography

## Abstract

Accurate detection and characterization of coronary atherosclerosis are essential for risk stratification and for guiding revascularization strategies. We aim to provide a pragmatic comparison of invasive and noninvasive imaging approaches for the detection, physiological assessment, and clinical management of coronary atherosclerosis. This review compares invasive techniques-coronary angiography, wire-based physiological assessment using fractional flow reserve (FFR) and instantaneous wave-free ratio, and intravascular imaging with intravascular ultrasound and optical coherence tomography - with noninvasive strategies including coronary artery calcium scoring, coronary computed tomography angiography (CCTA) with or without computed tomography - derived FFR, stress echocardiography, single-photon emission computed tomography, positron emission tomography (PET) with absolute myocardial blood flow quantification, and stress cardiovascular magnetic resonance imaging. We summarize diagnostic performance for anatomical plaque detection and ischemia assessment, strengths and limitations, patient selection considerations, safety issues related to radiation and contrast exposure, and downstream clinical impact. Invasive modalities remain the reference standard for lesion-level anatomical and physiological assessment when revascularization is contemplated. In contrast, noninvasive tests efficiently triage symptomatic patients, characterize plaque, and quantify ischemia and microvascular dysfunction. A pragmatic diagnostic algorithm integrates non-invasive anatomical or physiological testing as the initial step. It reserves invasive evaluation for high-risk findings or when percutaneous coronary intervention or coronary artery bypass grafting is likely. Future directions include refined plaque characterization on CCTA, quantitative PET perfusion imaging, computed tomography-derived FFR, and artificial intelligence-assisted image analysis.

## Introduction and background

Coronary atherosclerosis is the dominant substrate for ischemic heart disease worldwide; modern care depends on detecting plaque, distinguishing anatomical narrowing from lesion-level ischemia, and linking findings to prevention or revascularization. Rapid advances in invasive and noninvasive imaging now enable clinicians to visualize the coronary lumen and wall, phenotype plaque, and quantify myocardial perfusion/flow, reshaping diagnostic pathways and therapeutic decisions [1,2].

Large comparative trials clarified each modality's sweet spot. An anatomic strategy anchored in coronary computed tomography angiography (CCTA) expedites diagnosis and safely excludes obstructive disease in low- to intermediate-risk chest pain, while physiology-first strategies using stress imaging or invasive pressure indices (fractional flow reserve (FFR) or instantaneous wave-free ratio (iFR)) align revascularization with ischemia [3,4]. Simultaneously, outcome-focused work (e.g., Scottish Computed Tomography of the Heart Trial (SCOT-HEART), a randomized trial evaluating the addition of CCTA to standard care in patients with suspected stable angina) showed that simply identifying and reporting plaque, often non-obstructive, triggers intensified prevention and lowered myocardial infarction (MI) [5]. In stable disease, International Study of Comparative Health Effectiveness with Medical and Invasive Approaches (ISCHEMIA) [6] demonstrated that routine early revascularization does not reduce death or MI versus optimal medical therapy, strengthening a test strategy that (1) efficiently rules out obstructive coronary artery disease (CAD) when unlikely, (2) proves lesion-specific ischemia when percutaneous coronary intervention (PCI)/coronary artery bypass grafting (CABG) is considered, and (3) escalates prevention when plaque biology signals risk [1].

Within this framework, we compare invasive tools - invasive coronary angiography (ICA) for lumenography, FFR/iFR for lesion physiology, and intravascular ultrasound/optical coherence tomography (IVUS/OCT) for plaque/stent characterization - with noninvasive approaches spanning coronary artery calcium (CAC) scoring, CCTA (± computed tomography-derived fractional flow reserve (CT-FFR)), and functional imaging (stress echocardiogram, single-photon emission computed tomography (SPECT), positron emission tomography (PET) with myocardial blood flow (MBF)/coronary flow reserve (CFR), and stress cardiovascular magnetic resonance (CMR)) [6]. We synthesize diagnostic performance and practical trade-offs, then offer stepwise, pragmatic algorithms for common scenarios, including how to resolve anatomy-physiology discordance and when to escalate to invasive assessment. Finally, we highlight fast-moving areas - CT-FFR, quantitative PET flow, high-risk plaque on CCTA, and OCT markers of vulnerability/healing - and their likely impact on real-world pathways.

This article is a narrative review. A targeted literature search was performed using PubMed/MEDLINE, Scopus, and the Cochrane Library to identify relevant publications on invasive and non-invasive imaging for coronary atherosclerosis. The search included literature published from 2010 through 2025, with emphasis on contemporary studies and guideline documents. Search terms included combinations of the following keywords: coronary atherosclerosis, CAD, CCTA, CT-derived FFR, CT-FFR, stress echocardiography, single-photon emission computed tomography, SPECT, positron emission tomography, PET, MBF, CMR, ICA, FFR, iFR, IVUS, and OCT. Priority was given to major clinical trials, society guidelines, expert consensus statements, meta-analyses, and landmark observational studies with direct relevance to diagnostic accuracy, prognosis, clinical decision-making, and revascularization strategy.

## Review

Modalities at a glance: What they really tell us

The imaging toolbox for suspected or known coronary atherosclerosis has matured into two complementary streams: (1) invasive, lesion-level tools that can diagnose and treat in the same sitting, and (2) noninvasive tests that efficiently triage, phenotype plaque, and quantify ischemia with lower immediate risk (Table 1).

**Table 1:** Modality at a glance. ICA, invasive coronary angiography; FFR, fractional flow reserve; iFR, instantaneous wave-free ratio; IVUS, intravascular ultrasound; OCT, optical coherence tomography; CAC, coronary artery calcium; CCTA, coronary computed tomography angiography; CT-FFR, computed tomography-derived fractional flow reserve; Stress Echo, stress echocardiography; SPECT MPI, single-photon emission computed tomography myocardial perfusion imaging; PET, positron emission tomography; MBF, myocardial blood flow; CFR, coronary flow reserve; Stress CMR, stress cardiac magnetic resonance imaging; LGE, late gadolinium enhancement; PCI, percutaneous coronary intervention; ACS, acute coronary syndrome; LM, left main coronary artery; CAD, coronary artery disease; INOCA, ischemia with non-obstructive coronary arteries; ACC, American College of Cardiology; AHA, American Heart Association; ESC, European Society of Cardiology; SCCT, Society of Cardiovascular Computed Tomography; ASE, American Society of Echocardiography; ASNC, American Society of Nuclear Cardiology; SCMR, Society for Cardiovascular Magnetic Resonance; FAME, Fractional Flow Reserve versus Angiography for Multivessel Evaluation 2;  Functional Lesion Assessment of Intermediate Stenosis to Guide Revascularization; Instantaneous Wave-Free Ratio versus Fractional Flow Reserve in Patients with Stable Angina or Acute Coronary Syndromes (Swedish Web-System for Enhancement and Development of Evidence-Based Care in Heart Disease Evaluated According to Recommended Therapies); Multi-Ethnic Study of Atherosclerosis; PROMISE, Prospective Multicenter Imaging Study for Evaluation of Chest Pain; SCOT-HEART, Scottish Computed Tomography of the Heart Trial; NXT, Analysis of Coronary Blood Flow Using CT Angiography: Next Steps

Modality	Shows best	Unique strength	Key limitation	Typical use case	Anchor source
ICA	Lumen stenosis and distribution	Immediate PCI access	Underestimates plaque burden/composition	Definitive anatomy when revascularization is likely; ACS	ESC, ACC/AHA
IVUS	Plaque burden, remodeling, true size	Stent optimization; LM sizing	Lower microstructural resolution	Left main; diffuse disease; PCI optimization	IVUS consensus
OCT	Cap thickness, thrombus, stent detail	Near‑microscopic resolution	Shallow penetration; contrast flush	ACS culprit; stent malapposition/neointima	OCT consensus
FFR/iFR	Lesion‑specific ischemia	Outcome‑validated thresholds	Wire/adenosine (FFR); technical drift	Intermediate (40%-70%) lesions	FAME [7]
CAC	Calcified plaque burden	Primary prevention risk re‑stratification	No lumen stenosis/ischemia data	Asymptomatic risk refinement; gatekeeper	ACC/AHA; prevention
CCTA	Lumen + wall; high‑risk plaque	High sensitivity/NPV	Specificity ↓ with heavy calcification; contrast/radiation	Rule out obstructive CAD; phenotype plaque	PROMISE [8]; SCOT‑HEART [5]; SCCT
CT‑FFR	Per‑vessel physiology (non‑invasive)	Improves the specificity of CCTA	Needs high‑quality CCTA; vendor dep.	Equivocal 40%-90% stenoses on CCTA	NXT [9]; PLATFORM [3]; SCCT
Stress Echo	Inducible wall‑motion ischemia	No radiation; hemodynamics	Operator-/window-dependent	Functional test when contrast/radiation is undesired	ASE; ACC/AHA
SPECT MPI	Relative perfusion	Broad availability; prognostic data	Attenuation; lower spatial resolution	Triage and risk stratification	ASNC; ACC/AHA
PET (MBF/CFR)	Absolute flow and microvascular disease	Highest diagnostic accuracy; quantitative flow	Limited availability/cost	Multivessel/balanced ischemia; INOCA	ASNC/SCMR practice points
Stress CMR	Perfusion + scar (LGE)	No radiation; tissue characterization	Devices/claustrophobia; GBCA caution	High‑quality functional test; viability	MR‑INFORM [4]; SCMR

The art is fitting the clinical question-anatomy, physiology, or plaque biology-to the right stream and then integrating results so therapy tracks with outcomes.

Invasive angiography remains the interventional map, not the whole landscape. Contrast angiography outlines the lumen in exquisite temporal detail and shows where a stenosis pinches the channel. It is unrivaled for acute coronary syndromes (ACSs) and for stable patients in whom revascularization is already on the table. Yet classic luminography sees only the silhouette; positively remodeled or eccentric plaques can hide substantial disease, and visual percent stenosis correlates imperfectly with ischemia, especially in diffuse disease. Pitfalls, including foreshortening, vessel overlap, and calibration deviation, explain much of the *looks tight but isn't ischemic* phenomenon [1,7]. This is why modern catheterization practice routinely adds physiology.

Wire-based physiology (FFR/iFR) aligns PCI with ischemia. By comparing distal to aortic pressure under hyperemia (FFR) or during the diastolic wave-free period (iFR), we can ask: “Does this stenosis actually limit flow?” Randomized trials established two durable truths: (1) treating only ischemia-producing lesions improves outcomes versus angiography-guided stenting; (2) deferring non-ischemic lesions is safe. Thresholds (FFR ≤ 0.80; iFR ≤ 0.89) are simple, and iFR is non-inferior to FFR for decision-making [8-10]. Pullback maneuvers (pressure or imaging) further separate focal from diffuse gradients, avoiding futile *spot stents *in diffuse disease.

Intravascular imaging (IVUS/OCT) reveals the vessel you cannot see on angiography. IVUS measures true vessel size, plaque burden, and minimal lumen area; it is particularly helpful for the left main, long or calcified segments, and for improving expansion/apposition during PCI. OCT adds near-microscopic resolution, including fibrous cap thickness, lipid pool, calcium, thrombus, stent under-expansion, or malapposition, making it the modality of choice for ACS culprit interrogation and for stent failure mechanisms. The trade-offs are familiar: IVUS has lower resolution but deeper penetration; OCT requires contrast clearing and has shallow penetration. Together with physiology, intracoronary imaging converts ambiguity into mechanism-driven therapy [11].

CAC and CCTA shift anatomic mapping upstream. Outside the Catheterization lab, CAC quantifies calcified burden and upgrades prevention even when symptoms are absent; it is not a diagnostic test for obstructive CAD but a powerful preventive compass. CCTA then visualizes both lumen- and wall-detecting calcified and non-calcified plaque, as well as high-risk features (low attenuation, positive remodeling, napkin-ring sign, spotty calcification). In low- to intermediate-risk chest pain, the sensitivity and NPV are so high that CCTA is an excellent gatekeeper; trials show faster diagnosis, fewer non-diagnostic catheterizations, and improved preventive therapy when plaque is reported [12-13]. When stenoses fall in the 40%-90% gray zone or are obscured by heavy calcium, CT-derived FFR adds lesion-specific physiology without extra radiation, improving triage and avoiding needless ICA.

Functional imaging answers the physiology question noninvasively. Stress echocardiography offers portable, radiation-free detection of inducible wall-motion abnormalities and provides hemodynamic/valvular context; image quality and operator reliance are its limits [14]. SPECT myocardial perfusion imaging (MPI) remains globally available with solid prognostic data but is vulnerable to attenuation and has lower spatial resolution than newer options, which is important when multivessel disease produces balanced ischemia [15]. PET with absolute MBF and CFR overcomes those gaps: quantitation improves detection of multivessel and microvascular disease and provides potent incremental risk stratification across sex and risk strata [16,17]. Stress CMR integrates high-resolution, first-pass perfusion with late gadolinium enhancement (LGE) for scar/fibrosis, without ionizing radiation, and, in MR-INFORM (Magnetic Resonance INFORMing Management of Patients With Stable Coronary Artery Disease, a randomized trial evaluating stress cardiac magnetic resonance-guided management versus invasive FFR-guided management in stable coronary disease) [18], a CMR-first strategy was non-inferior to FFR-guided care for major events while yielding fewer revascularizations. Device/claustrophobia constraints and GBCA use (favor macrocyclic agents; caution in advanced chronic kidney disease (CKD)) are the practical boundaries.

Integration: The anatomy-to-physiology sequence

A contemporary pathway often starts with CCTA (± CT-FFR) to exclude obstructive disease and phenotype plaque in low-intermediate-risk patients; pivots to PET MBF/CFR or stress CMR when risk is higher, CCTA quality is limited (very high CAC, atrial fibrillation (AF) with high/irregular heart rate (HR)), or microvascular disease is suspected; and reserves ICA + FFR/iFR (± IVUS/OCT) for ACS, high-risk noninvasive findings, or when revascularization is truly contemplated. When anatomy and physiology conflict, physiology adjudicates revascularization, while any plaque, especially high-risk plaque on CCTA or vulnerable/stent-related pathology on OCT, should trigger aggressive prevention independent of percent stenosis [16,18].

Diagnostic performance

Test accuracy is not a fixed property of the modality, but a moving target determined by pretest probability, image quality, and the clinical endpoint you care about (anatomy vs. physiology). In symptomatic, low- to intermediate-risk patients, CCTA provides the best rule-out of obstructive CAD: per-patient sensitivity is typically ~95% to 99%, with a very high negative predictive value (NPV), when ≥50% stenosis on ICA is the reference. Specificity falls with heavy calcification or motion, which is exactly where adding CT-FFR helps, raising specificity for 40-90% lesions and cutting non-diagnostic catheterization without extra radiation [5,7].

When the clinical decision is revascularization, physiology is the right target. Invasive pressure-wire indices (FFR ≤0.80, iFR ≤0.89) outperform angiographic eyeballing; they reduce unnecessary stenting and safely defer non-ischemic lesions [19]. Among noninvasive options, stress PET and CMR offer the strongest association with lesion- or territory-level ischemia and prognosis. PET with absolute MBF and CFR improves detection of multivessel and microvascular disease and delivers powerful risk stratification beyond relative perfusion. CMR couples high-resolution perfusion with scar (LGE), and in MR-INFORM, a CMR-first strategy was non-inferior to FFR-guided care while yielding fewer revascularizations [16,18]. SPECT and stress echocardiography retain broad utility and mature prognostic data; performance depends on attenuation correction, stress-only protocols, and acoustic windows [14,15].

Two practical caveats frame interpretation. First, reported ranges (e.g., sensitivity/specificity) shift with prevalence, scanners, tracers, stressors, and analytic pipelines; per-patient figures (rule-in/rule-out) differ from per-vessel or per-lesion analyses [11]. Second, anatomy-physiology discordance is common: a tight-looking lesion may be non-ischemic (diffuse disease, remodeling), and ischemia may occur with non-obstructive plaque (microvascular dysfunction). In such conflicts, let physiology adjudicate revascularization, and let plaque detection (on CCTA or intracoronary imaging) trigger prevention escalation irrespective of percent stenosis [13].

Lean-on numbers (typical contemporary ranges, not absolutes):

· CCTA (≥50% stenosis): sensitivity ~95%-99%, NPV very high in low-intermediate risk; specificity variable with CAC/motion; CT-FFR area under the curve (AUC) ~0.80 to 0.90 and improves specificity for 40%-90% lesions [12].

· SPECT/Stress Echo: solid prognostic value; accuracy enhanced by attenuation correction (SPECT) and image quality (echo) [15].

· Stress PET (relative + MBF/CFR): high diagnostic performance vs. FFR; MBF/CFR strongly prognostic across sex/risk strata [16,17].

· Stress CMR: high diagnostic performance; MR-INFORM non-inferior to FFR-guided strategy for major adverse cardiovascular event (MACE) with fewer revascularizations [18].

· Invasive FFR/iFR: gold standard for lesion-level ischemia; physiology-guided PCI improves outcomes vs. angiography-guided care [19].

Advantages, limitations, and practicalities

Choosing an imaging strategy is less about identifying a single *best* test and more about matching the clinical question-anatomy, physiology, plaque biology, or procedural planning-to the patient in front of you. In practice, modality choice is shaped not only by diagnostic performance but also by rhythm, renal function, body habitus, prior revascularization, contrast tolerance, and local availability. Contemporary pathways, therefore, usually begin with noninvasive testing and escalate to invasive assessment when findings are high-risk, discordant, or likely to change revascularization decisions (Table 2) [2].

**Table 2:** Summary of practicalities and limitations of the different modalities. ICA, invasive coronary angiography; PCI, percutaneous coronary intervention; FFR, fractional flow reserve; iFR, instantaneous wave-free ratio; IVUS, intravascular ultrasound; OCT, optical coherence tomography; CAC, coronary artery calcium; CCTA, coronary computed tomography angiography; CT-FFR, computed tomography-derived fractional flow reserve; AF, atrial fibrillation; SPECT MPI, single-photon emission computed tomography myocardial perfusion imaging; PET, positron emission tomography; MBF, myocardial blood flow; CFR, coronary flow reserve; INOCA, ischemia with non-obstructive coronary arteries; Stress CMR, stress cardiac magnetic resonance imaging; LGE, late gadolinium enhancement; ACS, acute coronary syndrome; CKD, chronic kidney disease; NPV, negative predictive value; mSv, millisievert

Modality	Biggest advantages	Key limitations/contraindications	Typical radiation	Contrast	Where it shines
ICA	Definitive lumen; immediate PCI; add FFR/iFR and IVUS/OCT	Invasive risks; iodinated contrast; radiation; misclassification of intermediate lesions	~2 to 10 mSv	Iodinated	High-risk/discordant tests; when revascularization is likely
FFR/iFR	Lesion-specific physiology; outcomes-validated	Wire handling; adenosine for FFR; artifacts	Procedural	-	Intermediate (40%-70%) stenoses; PCI decision
IVUS	Plaque burden and vessel sizing; stent optimization	Lower microstructural detail vs OCT	Procedural	-	Left main/diffuse disease; complex PCI
OCT	Cap/thrombus; stent malapposition/neo-intima	Requires contrast flush; shallow penetration	Procedural	Iodinated (flush)	ACS culprit, stent troubleshooting
CAC	Fast, cheap, prognostic	No lumen/ischemia info; not for symptomatic diagnosis	~1 mSv	None	Primary prevention risk re-stratification
CCTA	Whole-tree anatomy; plaque phenotype; high NPV	↓Specificity with heavy calcium/AF; iodinated contrast	~1-5 mSv	Iodinated	Low-intermediate risk stable chest pain
CT-FFR	Adds physiology to CCTA; ↑specificity	Needs high-quality CCTA; vendor dependent	none (post-proc)	-	Equivocal 40%-90% lesions on CCTA
Stress Echo	No radiation; hemodynamics/valves	Operator and window dependent	none	none	Functional triage when contrast/radiation is undesired
SPECT MPI	Broad access; robust prognostic data	Attenuation; lower spatial resolution	~6-12 mSv	Radiotracer	General functional testing
PET (MBF/CFR)	Highest accuracy; absolute flow; microvascular detection	Availability/cost; tracer logistics	~2-5 mSv	Radiotracer	Multivessel/balanced ischemia; INOCA
Stress CMR	No radiation; perfusion + scar (LGE)	Devices/claustrophobia; gadolinium caution in severe CKD	none	Gadolinium	Tissue characterization; viability; high-quality perfusion

Invasive Strategies

Invasive strategies are most useful when lesion-level decision-making and immediate treatment are both on the table. ICA remains the procedural reference standard because it provides rapid lumen assessment and enables same-sitting intervention when needed. Pressure-wire physiology adds the crucial question that angiography alone cannot answer: whether a given stenosis is actually flow-limiting. In this setting, FFR and iFR align PCI with ischemia, reduce unnecessary stenting, and allow safe deferral of non-ischemic lesions. Intravascular imaging then adds mechanism and precision [10,19]. IVUS is particularly valuable for vessel sizing, plaque burden assessment, and optimization of left main, diffuse, or calcified PCI, whereas OCT provides near-microscopic detail for thrombus, fibrous-cap disruption, tissue protrusion, stent under-expansion, malapposition, and other culprit or stent-failure mechanisms.

The major strength of invasive assessment is therefore not simply that it *shows the artery*, but that it can connect anatomy, physiology, and treatment within a single workflow. Its limitations are equally important. Luminography alone can underestimate disease in positively remodeled or eccentric plaques and correlates imperfectly with lesion-level ischemia. Invasive testing also carries procedural risk, including vascular complications, stroke, acute kidney injury, radiation exposure, and contrast burden. For this reason, routine ICA is best reserved for patients in whom revascularization is likely, ACS is suspected, or noninvasive testing is high-risk, indeterminate, or discordant in a way that would alter care [20].

In practical terms, invasive strategies should be used deliberately rather than reflexively. Intermediate lesions should generally undergo physiological assessment, and pullback maneuvers can help distinguish focal from diffuse gradients before “spot stenting” diffuse disease. IVUS should be favored when vessel sizing and expansion are central, particularly in left main, diffuse, or calcified segments, whereas OCT is especially useful when culprit morphology or stent pathology will determine strategy. In patients with CKD or prior contrast-associated kidney injury, contrast-sparing approaches, including IVUS-guided low-contrast PCI, should be considered whenever invasive treatment is necessary [10].

Noninvasive Strategies

Noninvasive strategies span a broader diagnostic range. They can exclude obstructive CAD, define plaque phenotype, measure ischemic burden, estimate prognosis, and identify diffuse or microvascular dysfunction before a catheter is ever opened. CCTA is particularly effective in low-intermediate-risk chest pain because of its high sensitivity and NPV, while also providing plaque-level information that sharpens preventive care [13,21]. When stenoses fall into the 40%-90% range, or CCTA leaves uncertainty, CT-derived FFR improves specificity and helps avoid non-diagnostic catheterization. Functional modalities answer a different question: whether ischemia is present and how extensive it is. PET with MBF/CFR and stress CMR provide the strongest noninvasive physiological characterization when available, while SPECT and stress echocardiography remain pragmatic and guideline-supported options in many real-world settings [15,16].

CCTA specificity declines with heavy calcification or fast/irregular rhythms; beta-blockade/nitroglycerin and protocol optimization matter. PET/CMR access can be limited; CMR needs a gadolinium-based contrast agent (GBCA; caution in advanced CKD). SPECT has lower spatial resolution and is prone to attenuation; stress echocardiography is operator-dependent. A positive noninvasive test still requires ICA if revascularization is planned [15,16].

The strengths of noninvasive imaging are efficiency, lower immediate procedural risk, and the ability to triage patients before invasive treatment is contemplated. Just as important, noninvasive tests often define the downstream pathway: a normal high-quality CCTA may end the ischemic workup, whereas a high-risk functional study may justify ICA with lesion-specific physiology. Their limitations, however, are modality-specific. CCTA becomes less specific in the setting of heavy calcification or fast/irregular rhythms. PET and CMR may be constrained by access, expertise, and cost. CMR requires gadolinium-based contrast when perfusion and scar characterization are pursued, which raises caution in advanced CKD [15, 16]. SPECT has lower spatial resolution and is vulnerable to attenuation artifacts, while stress echocardiography is more dependent on acoustic windows and operator skill. Importantly, a positive noninvasive test does not itself replace ICA when revascularization is being considered; it usually serves as the gatekeeper to invasive confirmation and treatment planning [2].

From a practical standpoint, test selection should remain question-driven. CCTA is typically the first-line anatomic test for low-intermediate-risk stable chest pain. In intermediate-high-risk patients, those with known CAD, or those in whom physiology is the core question, PET MBF/CFR or stress CMR often provide more useful information. SPECT and stress echocardiography remain important when resources, logistics, or contraindications limit access to PET or CMR. When anatomy and physiology disagree, ischemia rather than percent stenosis should adjudicate revascularization decisions.

Special Populations and Patient-Specific Modifiers

In clinical practice, the selection of an imaging modality is often determined less by theoretical diagnostic performance and more by patient-specific factors that influence image quality, safety, and interpretability. Renal function, cardiac rhythm, coronary calcification, prior revascularization, body habitus, and device compatibility can all shift the balance between an anatomic-first and physiology-first strategy. Recognizing these modifiers early allows clinicians to select the most appropriate test while avoiding technically limited or non-diagnostic studies [12,22].

Advanced CKD and contrast limitation: In patients with advanced CKD or prior contrast-associated acute kidney injury, the use of iodinated contrast for CCTA or ICA should be carefully weighed against potential renal risk. In such settings, stress echocardiography or stress CMR may provide safer initial alternatives, with gadolinium-based contrast used cautiously when CMR is pursued and the anticipated diagnostic benefit outweighs potential harm. When invasive evaluation is required, contrast-sparing strategies, meticulous hydration, and intravascular imaging-guided, low-contrast PCI may help mitigate renal injury [2,22-23].

Prior stents, heavy calcification, and prior CABG: Patients with multiple prior stents, severe coronary calcification, or prior CABG often present challenges for an anatomic-first strategy. Blooming artifact from stents and dense calcification can reduce the specificity of CCTA and overestimate stenosis severity, while complex graft anatomy may further limit interpretability. In these cases, functional imaging with PET MBF/CFR or stress CMR is often more informative when the clinical question centers on ischemia. When revascularization is being considered, ICA with lesion-specific physiology and adjunctive intravascular imaging (IVUS or OCT) remains the most reliable approach [22,24]. 

AF and fast or irregular heart rates also reduce CCTA image quality and often shift testing toward PET or stress CMR. Large body habitus and attenuation artifacts may lower confidence in SPECT or stress echocardiography, making PET or CMR more robust alternatives when available. Likewise, implants, claustrophobia, or pregnancy may constrain CMR and favor echocardiography or carefully selected non-contrast approaches. Finally, in patients with persistent ischemic symptoms despite non-obstructive epicardial findings, the workup should not stop; PET MBF/CFR or stress CMR should be used to evaluate diffuse ischemia or microvascular dysfunction rather than concluding prematurely that clinically relevant coronary disease is absent [25-28].

Safety Considerations

Safety should be considered alongside diagnostic yield at the time of test selection, not after the fact. Radiation exposure varies meaningfully across modalities: CAC scoring is typically low dose, CCTA is often in the low-to-moderate range depending on protocol, PET is generally lower dose than SPECT, and diagnostic ICA adds procedural radiation exposure; stress CMR and stress echocardiography avoid ionizing radiation entirely. Dose-reduction strategies such as prospective ECG gating, tube-current modulation, lower kVp in smaller patients, high-pitch or wide-detector CT protocols, and stress-only SPECT, where appropriate, should be part of routine imaging stewardship.

Contrast-related safety is equally important. Iodinated contrast used in CCTA, ICA, and OCT requires attention to renal function, hydration status, and avoidance of unnecessary nephrotoxic exposures. Gadolinium use in CMR should favor macrocyclic agents, with particular caution in advanced CKD and consideration of non-contrast protocols where feasible. Radiotracers used in SPECT and PET are not nephrotoxic, but they do contribute to cumulative radiation exposure. At a practical level, the lowest-risk test is the one that answers the clinical question adequately without creating avoidable downstream harm [29].

Taken together, these practicalities reinforce a simple principle: start with the modality that best answers the relevant clinical question at the lowest reasonable risk, escalate to invasive physiology when revascularization is genuinely being considered, and use intravascular imaging when procedural precision will change management.

Clinical pathways and decision algorithms

Clinicians choose between noninvasive and invasive testing by anchoring the decision to the question at hand: Do we need anatomy, physiology, or plaque biology? They also weigh pretest probability, patient factors, and whether revascularization is realistically on the table. In most stable positions, the safest and most informative opening move is noninvasive. For patients with low-intermediate likelihood of obstructive CAD, CCTA) rapidly maps the lumen and vessel wall with high sensitivity and NPV, and it phenotypes plaque in ways that sharpen preventive care. When CCTA reveals a 40%-90% stenosis or image uncertainty, adding CT-derived FFR raises specificity and averts non-diagnostic trips to the Catheterization lab, a pathway that has repeatedly shortened time to diagnosis and improved downstream therapy [20,28-31]. If CCTA quality is likely to suffer - because of very high calcium burden or an irregular/fast rhythm - physiology becomes the better first answer, with stress CMR or PET MBF/CFR preferred for their accuracy and prognostic strength [12].

**Figure 1:**
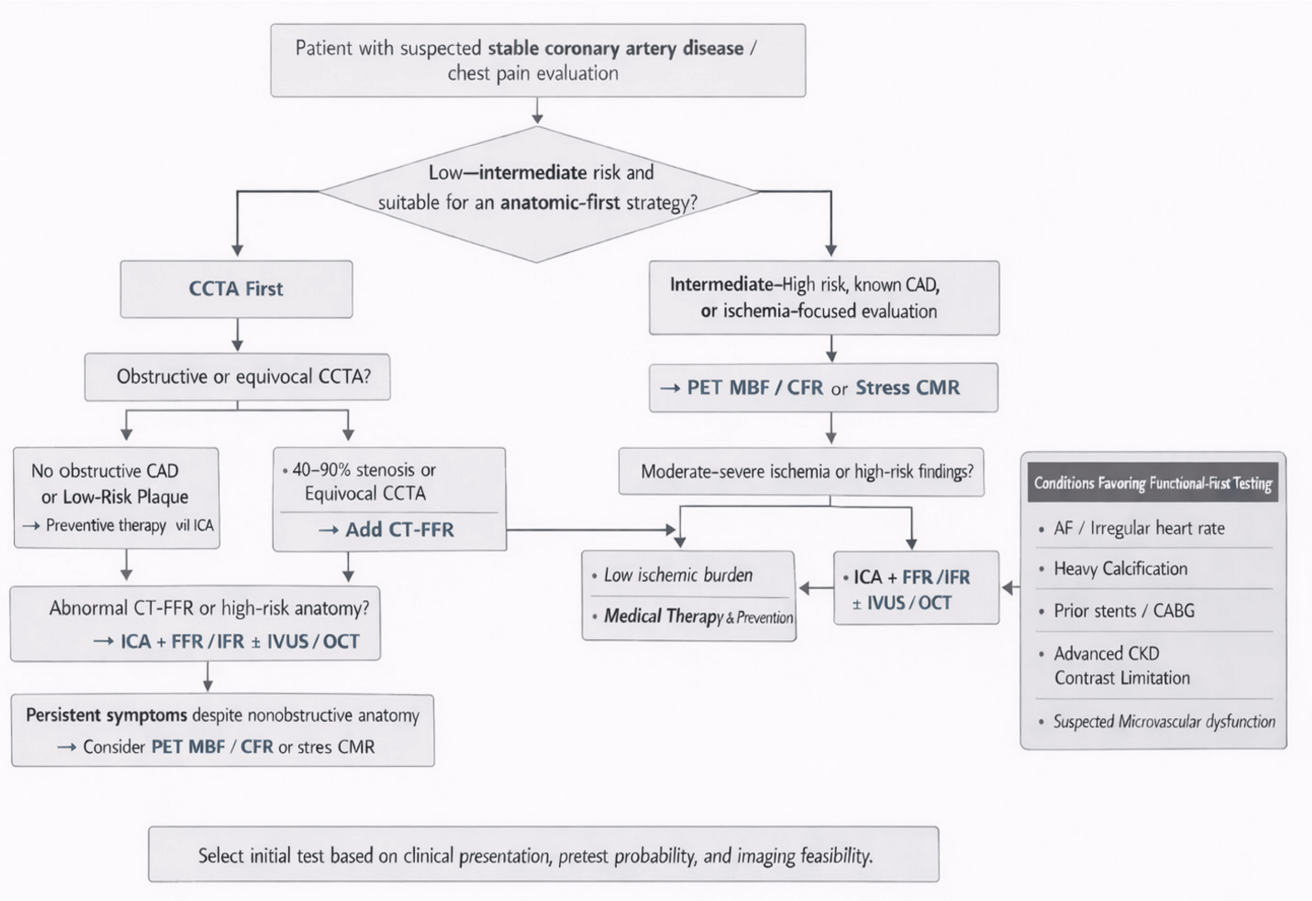
Pragmatic coronary imaging pathway: choosing an anatomic-first versus functional-first strategy. In patients with low-intermediate pretest probability and new stable chest pain, an anatomic-first strategy with CCTA is generally preferred because of its high sensitivity and NPV. When CCTA shows 40-90% stenosis or is technically uncertain, CT-FFR improves specificity and helps determine whether ICA is needed. In patients with intermediate-high risk, known CAD, or when ischemia is the dominant clinical question, a functional-first strategy with PET MBF/CFR or stress CMR is preferred, with SPECT MPI or stress echocardiography as pragmatic alternatives where access dictates. If revascularization is likely to change management, ICA with FFR/iFR and adjunctive IVUS/OCT is appropriate. CCTA limitations such as AF, heavy calcification, prior revascularization, advanced CKD, or suspected microvascular dysfunction may favor a physiology-first pathway. Image created by the authors using Microsoft PowerPoint (Microsoft Corporation, Redmond, WA). CCTA, coronary computed tomography angiography; NPV, negative predictive value; CT-FFR, computed tomography-derived fractional flow reserve; ICA, invasive coronary angiography; CAD, coronary artery disease; PET, positron emission tomography; MBF, myocardial blood flow; CFR, coronary flow reserve; CMR, cardiovascular magnetic resonance; SPECT MPI, single-photon emission computed tomography myocardial perfusion imaging; FFR, fractional flow reserve; iFR, instantaneous wave-free ratio; IVUS, intravascular ultrasound; OCT, optical coherence tomography; AF, atrial fibrillation; CKD, chronic kidney disease

When the clinical likelihood is high, symptoms are refractory/high-risk, or a change in therapy is expected, invasive angiography becomes appropriate-but it should be paired with lesion-level physiology. FFR aligns PCI with ischemia rather than appearance, while intravascular imaging provides the mechanistic detail that angiography alone cannot: IVUS for vessel sizing and expansion in left main or diffuse disease, and OCT when cap disruption, thrombus, or stent pathology will change the plan [32]. This philosophy is consistent with trials showing that, in stable disease, routine early revascularization does not reduce death or MI compared with optimal medical therapy, even as angina often improves, hence the emphasis on physiology to target who actually benefits and safely defer non-ischemic lesions. The overall strategy for selecting an anatomic-first versus functional-first imaging approach is summarized in Figure 1.

ACSs are different: here, invasive angiography is the entry point for culprit identification and PCI. For non-culprit intermediates, physiology again outperforms eyeballing severity, and OCT can adjudicate plaque morphology when it will alter antithrombotic strategy or stenting. Evidence supports complete revascularization of significant non-culprit lesions during the index stay or planned, although FLOWER-MI (Flow Evaluation to Guide Revascularization in Multivessel ST-Elevation Myocardial Infarction, a randomized trial comparing FFR-guided versus angiography-guided complete revascularization in ST-elevation myocardial infarction (STEMI) patients with multivessel disease) prompted debate about routine FFR guidance for every non-culprit lesion in STEMI [14,33-34].

Prior revascularization requires tailoring. With prior stents, blooming can limit CCTA, so recurrent angina typically starts with functional imaging (PET/CMR; SPECT/echocardiography as available). If revascularization is contemplated, ICA with FFR/iFR adjudicates significance, OCT clarifies mechanisms of stent failure, and IVUS secures sizing and expansion. For prior CABG, selective CCTA mapping of grafts can help when it will change care, but physiology with PET/CMR often guides whether redo revascularization is warranted; invasive angiography follows when a change in therapy is likely or noninvasive testing is high-risk [35-36].

Across scenarios, patient factors frequently redirect the route. CKD and contrast allergy push the plan toward stress echocardiography or CMR (with macrocyclic GBCA used carefully in advanced CKD) or toward minimal-contrast PCI using IVUS when the catheterization laboratory is necessary [27]. AF or high/irregular HR degrades CCTA and favors PET or CMR. Heavy calcification lowers CCTA specificity and nudges clinicians to physiology (PET/CMR or ICA + FFR/iFR). Large body habitus and attenuation artifacts tend to favor PET or CMR over SPECT/echocardiography. Implants, claustrophobia, or pregnancy may preclude gadolinium CMR and favor echocardiography or carefully selected non-contrast CMR [25-28,35].

A practical way to operationalize this is simple: estimate pretest probability; start with CCTA in low-intermediate risk and add CT-FFR when stenosis is 40%-90%; move to ICA with physiology (plus IVUS/OCT when needed) for high likelihood, high-risk findings, or treatment-deciding uncertainty; and, when symptoms continue despite non-obstructive coronaries, evaluate microvascular disease with PET MBF/CFR or stress CMR and tailor anti-anginal and preventive therapy accordingly [3,7,22]. Throughout a health system, a CCTA-first pathway in appropriate patients reduces non-diagnostic angiography and costs, while PET/CMR provides the highest diagnostic and prognostic value where accessible; SPECT and stress echocardiography remain pragmatic and guideline-supported alternatives. Standardized reporting (e.g., Coronary Artery Disease-Reporting and Data System (CAD-RADS) 2.0) and clear downstream rules keep the whole pathway consistent and auditable [30,36,37].

Pragmatic diagnostic algorithm

Modern care pathways start by aligning with the clinical question: rule out disease, localize ischemia, plan revascularization, or intensify prevention based on pretest probability and real-world limitations. A quick pre-test checklist helps: clarify the goal; review history/exam (age, sex, symptom quality, risk factors, prior PCI/CABG, current prevention); obtain ECG and basic labs (including renal function if contrast is possible); and screen logistics/contraindications that steer modality choice (implants/claustrophobia for CMR, contrast allergy/CKD for CT/ICA, rhythm and rate control for CCTA) [20]. A summary of the pragmatic algorithm is given in Table 3.

**Table 3:** Pragmatic algorithm/quick pathway table. CCTA, coronary computed tomography angiography; CT-FFR, computed tomography-derived fractional flow reserve; ICA, invasive coronary angiography; PET, positron emission tomography; CMR, cardiac magnetic resonance imaging; SPECT, single-photon emission computed tomography; FFR, fractional flow reserve; iFR, instantaneous wave-free ratio; IVUS, intravascular ultrasound; OCT, optical coherence tomography; CAD, coronary artery disease

Clinical scenario	First-line test	If obstructive/uncertain	When to perform ICA
Low-intermediate risk, new chest pain	CCTA	CT-FFR for 40%-90% stenosis or limited CCTA	When revascularization is likely (symptoms, ischemia, high-risk anatomy)
Intermediate-high risk or known CAD	Functional imaging (PET/CMR; SPECT/Echo as available)	Ischemia/high-risk → ICA + FFR/iFR (± IVUS/OCT)	After a positive/high-risk functional test or persistent symptoms
Discordant anatomy vs. physiology	-	Prioritize physiology	Use ICA + FFR/iFR when decisions hinge on lesion-level ischemia

Low-Intermediate Risk: New Chest Pain

CCTA is usually the first test because it rapidly maps the lumen and wall with high NPV. Reports should be structured (e.g., CAD-RADS 2.0) to convey stenosis severity, plaque phenotype, and future management [7,28,32]. When CCTA shows 40%-90% stenosis or image uncertainty, add CT-derived FFR to raise specificity and avoid nondiagnostic catheterizations; CT perfusion is an alternative where available. Move to invasive angiography when revascularization is likely (ischemia present, symptoms despite therapy, or high-risk anatomy such as left main/proximal multivessel) or when noninvasive results are non-diagnostic yet decision-critical [20]. In stable chest pain, CCTA has also proven a safer gatekeeper than routine ICA, with fewer major complications [38,39]. Operationally, slow the heart to <60-65 bpm and give sublingual nitroglycerin to optimize image quality; in very high calcium scores, pivot to PET or CMR for physiology rather than forcing CT specificity.

Intermediate-High Risk or Known CAD

Here, physiology answers the key question. Prefer PET with absolute MBF/CFR or stress CMR when available, as both provide strong diagnostic/prognostic separation and detect diffuse or microvascular disease; SPECT or stress echocardiography remain guideline-endorsed, widely accessible options [18]. CMR pairs high-resolution perfusion with scar characterization and, in MR-INFORM, was non-inferior to an FFR-guided invasive strategy for major outcomes. If testing shows moderate-severe ischemia or other high-risk features, proceed to ICA with lesion-specific physiology (FFR ≤ 0.80 or iFR ≤ 0.89) and use IVUS/OCT for sizing, plaque/stent assessment, and PCI optimization [10,40]. Outcome trial ISCHEMIA reinforces the principle: routine early revascularization does not lower death or MI versus optimal medical therapy in stable disease, so physiology should guide PCI while prevention is intensified for all [41].

Handling Discordant Results (Anatomy vs. Physiology)

**Figure 2:**
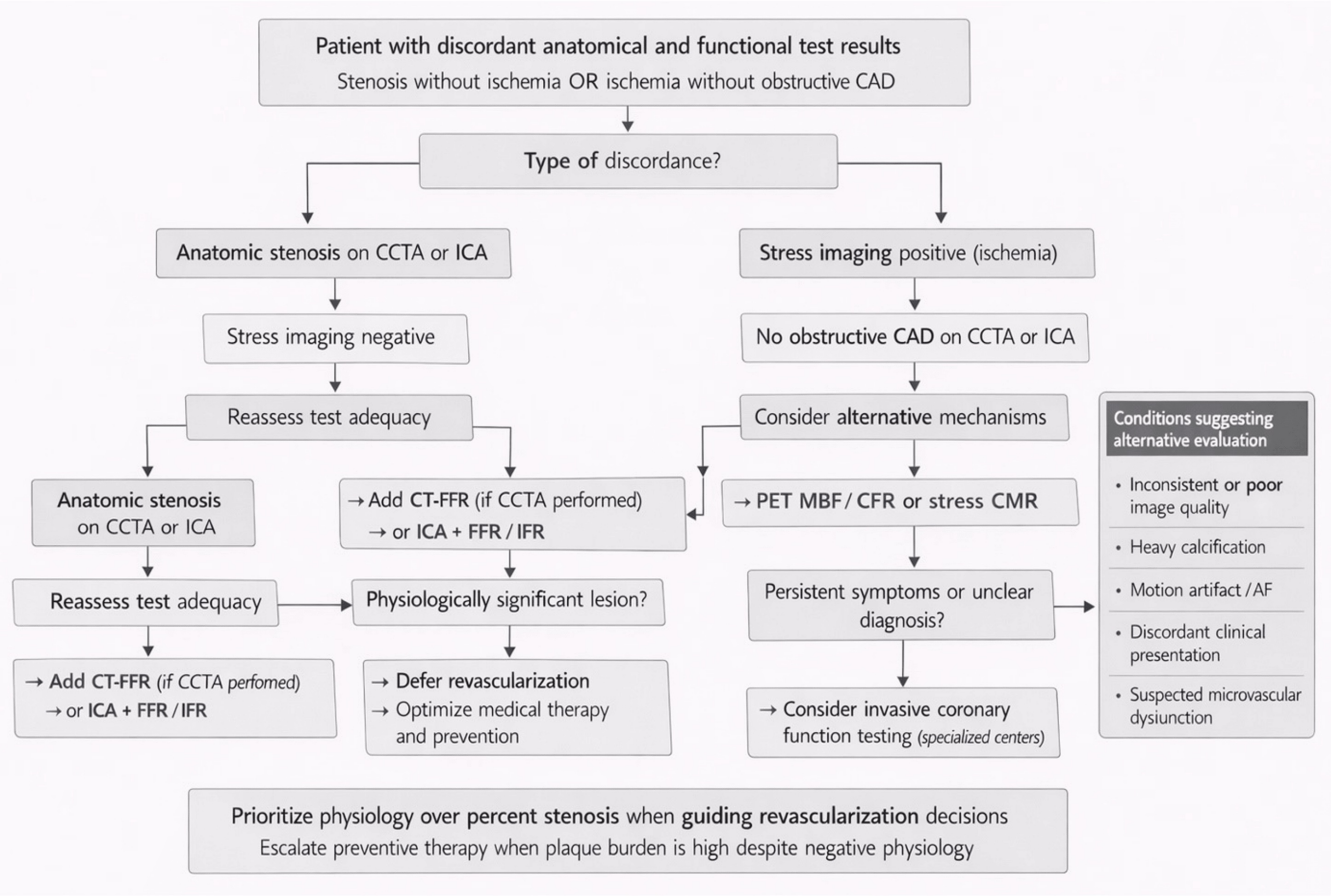
Practical approach to anatomy-physiology discordance in coronary imaging. Discordance between anatomical stenosis and ischemic findings is common and should be resolved by prioritizing physiological significance over percent stenosis alone when revascularization decisions are being considered. When anatomical imaging suggests significant disease but functional testing is negative, lesion-level physiology using CT-FFR or invasive FFR or iFR should be used to determine ischemic relevance. Conversely, when ischemia is present despite non-obstructive epicardial coronary arteries, clinicians should consider microvascular dysfunction, diffuse atherosclerosis, or vasomotor abnormalities, with PET MBF/CFR or stress CMR providing further characterization. In selected patients, invasive coronary function testing may be considered. Equivocal or technically limited studies should prompt targeted repeat or alternative testing based on the missing physiological or anatomical information. Image created by the authors using Microsoft PowerPoint (Microsoft Corporation, Redmond, WA). CAD, coronary artery disease; CCTA, coronary computed tomography angiography; ICA, invasive coronary angiography; CT-FFR, computed tomography-derived fractional flow reserve; iFR, instantaneous wave-free ratio; PET, positron emission tomography; MBF, myocardial blood flow; CFR, coronary flow reserve; CMR, cardiac magnetic resonance

A practical approach to resolving anatomy-physiology discordance is illustrated in Figure 2.

Adverse plaque on CCTA may not cause ischemia, and significant ischemia can occur without tight epicardial stenosis (e.g., diffuse or microvascular disease). When decisions hinge on revascularization, prioritize physiology (CT-FFR, PET/CMR, or invasive FFR/iFR), because outcomes track lesion-level ischemia better than percent stenosis. Practical steps include confirming image quality and adequacy of stress; add CT-FFR after CCTA if not yet performed; escalate to ICA + physiology when uncertainty persists, and treatment would change. If physiology is negative but plaque burden/adverse features are high, intensify prevention and monitor-an approach associated with lower MI in CCTA-first pathways [42-43]. When symptoms persist despite non-obstructive coronary arteries, evaluate for microvascular disease using PET MBF/CFR or stress CMR, and consider invasive coronary function testing in experienced centers [44].

Suspected INOCA/Microvascular Dysfunction

A normal or non-obstructive epicardial coronary study does not end the ischemic workup in patients with persistent angina or objective evidence of ischemia. Ischemia with non-obstructive coronary arteries (INOCA) should be suspected when symptoms are disproportionate to angiographic findings, when stress testing is abnormal despite non-obstructive coronaries, or when angina persists after apparently adequate treatment of epicardial CAD. In this setting, PET with absolute MBF and CFR is the preferred noninvasive quantitative test because it detects diffuse and microvascular flow impairment that relative perfusion imaging or angiography may miss [45]. Stress CMR is a valuable complementary option, particularly when PET is unavailable or when simultaneous assessment of perfusion, scar, and alternative myocardial pathology is desired [46]. In selected patients with persistent symptoms or unresolved diagnostic uncertainty, invasive coronary function testing using thermodilution- or Doppler-based assessment of microvascular function should be considered in experienced centers [47]. The key practical implication is that normal epicardial anatomy does not exclude ischemic heart disease; rather, it should prompt a shift from lesion detection to physiologic characterization [48].

Contemporary care works best when anatomy and physiology aren’t treated as rivals but as a relay team. In patients with low-intermediate pretest likelihood, CCTA is the natural lead-off: it rapidly maps the entire coronary tree, profiles plaque, and - because of its high sensitivity and NPV - safely rules out obstructive disease. When stenosis falls in the murky 40%-90% range or image quality is imperfect, adding CT-derived FFR tightens specificity and prevents nondiagnostic trips to the Catheterization lab. For patients with intermediate-high risk or established CAD, physiology should take the baton: PET with absolute MBF and CFR, or stress CMR, best quantify ischemia (including diffuse and microvascular disease) and provide powerful prognosis; SPECT and stress echocardiography remain pragmatic where access dictates. If revascularization is likely, invasive angiography paired with lesion-specific physiology (FFR/iFR) aligns stenting with ischemia, while IVUS/OCT refines sizing, expansion, and culprit characterization-an anatomy-to-physiology sequence that corresponds to guidelines and trial data and improves appropriateness and outcomes [20,28,38,49-50].

In practice, the first question is always: “What do I need to know-anatomy, physiology, or both?” When the goal is to exclude disease and plaque in low-to-intermediate-risk chest pain, CCTA (reported with CAD-RADS 2.0) is preferred; equivocal segments or intermediate stenoses benefit from CT-FFR. Conversely, when the goal is to localize ischemia and assess its burden in intermediate- to high-risk patients or those with known CAD, PET MBF/CFR or stress CMR should take precedence, with SPECT/echocardiography as accessible stand-ins. Revascularization decisions should be grounded in physiology: FFR ≤ 0.80 or iFR ≤ 0.89 so that non-ischemic lesions are safely deferred, and ischemia-producing lesions are treated. When anatomy and physiology disagree, re-examine image quality, add CT-FFR if appropriate, and let ischemia adjudicate; if physiology is negative but plaque burden or adverse features are high, escalate prevention rather than stenting [48,51].

Catheter-based imaging is most impactful when it changes what you do next. IVUS is the workhorse for left main and diffuse disease, vessel sizing, and expansion indices; OCT shines when cap disruption, thrombus, calcified nodules, or stent failure mechanisms will alter antithrombotic strategy or stent technique. Used thoughtfully, these tools convert ambiguity into implementable steps-better expansion, fewer edge problems, clearer culprit identification [10].

Patient factors often redirect the pathway. CKD or prior contrast-associated AKI favors echocardiography or CMR (with cautious use of macrocyclic GBCA in advanced CKD) and minimal-contrast strategies if proceeding invasively. AF or fast, irregular HRs reduce CCTA quality and push toward PET/CMR. Heavy calcification lowers CCTA specificity and shifts care toward functional testing or invasive physiology if revascularization is being considered. In patients with large body habitus or challenging attenuation, PET or CMR typically outperforms SPECT/echocardiography [22,39,52,53].

Clear reporting and quality metrics keep the pathway reproducible. CCTA reports should include CAD-RADS, segment-level stenosis, plaque phenotype, limitations, and the recommended next step (e.g., CT-FFR vs. functional testing). CT-FFR should provide per-lesion values with technical confidence notes. Functional studies must state stress adequacy, ischemic burden by territory, LV function, and key artifacts; PET adds MBF/CFR and thresholds, and CMR specifies perfusion method and LGE patterns. In the Catheterization lab, record FFR/iFR values and pullback (focal vs. diffuse) alongside the final decision rationale, and for IVUS/OCT, document vessel size, minimum lumen area, expansion/apposition, cap/thrombus/tissue protrusion, and exactly how the findings altered management [22,40,50-54].

Finally, systems should track whether testing is guideline-concordant, how often CCTA avoids nondiagnostic ICA (and how much CT-FFR improves that yield), and whether PCI is performed with documented ischemia. Time-to-diagnosis, 90-day revisits, and one-year MACE by pathway tie imaging choices to outcomes, while radiation and contrast dashboards reinforce stewardship (e.g., low-dose CT protocols, stress-only SPECT where appropriate). The evidence base is strong, but gaps remain, particularly head-to-head PET vs. CMR and the impact of AI plaque quantification and CT-FFR at scale on outcomes, so programs should maintain adaptability as new data arrive [36,55].

Emerging and future directions

The next phase of coronary imaging is less about finding a tighter lumen and more about reading plaque biology, microvascular health, and flow, then using that information to change outcomes. On CCTA, the story already extends well beyond percent stenosis: low-attenuation plaque, positive remodeling, napkin-ring morphology, and spotty calcification consistently track with future events, independent of luminal narrowing. When these features cluster, risk rises; when we report them clearly, clinicians intensify prevention: lipids, antiplatelets, lifestyle, and patients benefit. Quantified plaque metrics (total plaque, non-calcified, and low-attenuation volumes) provide even finer risk granularity and may be more sensitive than stenosis for monitoring response to therapy [21,56].

Physiology is evolving, too. PET with absolute MBF and CFR turns diffuse atherosclerosis and microvascular dysfunction from *invisible* to measurable. Because CFR integrates epicardial and microvascular resistance, it predicts events even when angiography is non-obstructive, helping explain symptoms and guiding therapy (anti-anginals, risk-factor modification) across sex and risk strata. In short, quantifying flow and prognosis sharpens [57,58].

After an anatomic test, precision matters. CT-derived FFR raises specificity for 40%-90% lesions and consistently trims nondiagnostic catheterizations in clinical practice pathways, where motion, calcium, or borderline segments cloud interpretation; CT perfusion (static or dynamic) can supply direct perfusion data. Protocols and doses differ by vendor, but the direction of travel is clear: smarter CT pipelines that couple plaque phenotype with physiology [42,43].

Software is catching up with biology. AI tools now automate calcium scoring, lumen/outer-wall segmentation, and plaque quantification (including low-attenuation and fibro-fatty volumes). Early multimodal models that fuse AI-derived plaque metrics with CT-FFR and clinical variables outperform standard risk scores for MACE prediction and suggest faster, more standardized reporting. We still need prospective, randomized evidence of impact on outcomes, but adoption in reading rooms is moving quickly [24].

Hybrid imaging tightens the loop between structure and function. PET/CT aligns plaque phenotype with quantitative flow in a single sitting, and investigational tracers such as ¹⁸F-NaF visualize active microcalcifications linked to vulnerability. PET/MR pairs quantitative PET flow with fine-detail CMR perfusion, edema, and scar - an ischemia-viability workup with lower radiation exposure, though availability remains concentrated in specialized hubs [59].

Inside the artery, OCT pushes precision care in ACS and stent failure. With near-microscopic resolution of cap thickness, thrombus, and calcified nodules, OCT can separate rupture from erosion (shaping stenting and antithrombotic intensity), explain stent failure (under-expansion, malapposition, neoatherosclerosis), and, on serial imaging after ACS, document cap increasing with intensive lipid therapy-supporting tailored secondary prevention and possibly informing dual antiplatelet therapy duration. Standardized acquisition/analysis now enables multi-center studies [60].

Translational Direction

The through-line is implementation: routine reporting of high-risk CCTA plaque features; wider adoption of PET flow quantitation where feasible; selective CT-FFR/CT perfusion after CCTA to avoid nondiagnostic catheterizations; and targeted OCT use in ACS and stent failure. Fold these advances into guideline-concordant prevention and physiology-guided revascularization, and the field moves from better pictures to better outcomes [61].

AI-driven CCTA analysis is moving from proof of concept toward workflow integration. Current platforms can automate coronary calcium scoring, coronary segmentation, plaque burden quantification, and identification of high-risk plaque phenotypes such as low-attenuation plaque and positive remodeling. These tools may reduce inter-observer variability, shorten reporting time, standardize plaque measurement across readers, and improve risk stratification when combined with clinical variables and CT-derived physiology. In practice, the near-term role of AI is likely to be as a decision-support layer that augments - not replaces - expert interpretation, particularly in high-volume CCTA services. Prospective studies are still needed to show that AI-guided plaque quantification improves downstream clinical outcomes, not only reporting efficiency [59,62-69].

## Conclusions

Contemporary cardiovascular care is most effective when anatomical and physiological assessments are integrated rather than viewed as competing strategies. In patients with low- to intermediate-risk chest pain, CCTA serves as an efficient first-line modality, offering high sensitivity for excluding obstructive CAD while simultaneously characterizing atherosclerotic plaque and informing preventive strategies. When anatomical findings are uncertain, adjunctive techniques such as CT-FFR improve diagnostic specificity and reduce unnecessary ICA procedures. In patients with higher-risk clinical profiles or established CAD, physiological assessment assumes greater importance, with PET and stress CMR providing a comprehensive evaluation of ischemia, including coronary microvascular dysfunction. When revascularization is being considered, ICA combined with lesion-specific physiological assessment ensures that intervention is directed toward ischemia-producing lesions, while IVUS further refines procedural planning and optimization.

Ultimately, the optimal diagnostic pathway is question-driven and patient-specific, requiring careful consideration of clinical presentation, comorbidities, local expertise, and the strengths and limitations of each imaging modality. Noninvasive testing should be used strategically to guide downstream decision-making, reserving invasive evaluation for circumstances in which results are expected to alter management. Discordance between anatomical stenosis and physiological significance should generally be resolved in favor of ischemia-guided decision-making, while the identification of high-risk plaque should prompt aggressive preventive therapy regardless of stenosis severity. As imaging technologies continue to evolve, including advances in quantitative flow assessment, plaque characterization, and artificial intelligence, the future of coronary evaluation will increasingly emphasize precision, integration, and outcome-driven care pathways.

Several limitations of this review should be acknowledged. As a narrative review, the literature selection was non-systematic and therefore subject to potential author selection bias. Formal quality assessment of included studies was not performed, and some conclusions rely on evidence synthesized across heterogeneous study designs. Furthermore, the rapidly evolving nature of technologies such as AI-based plaque quantification and CT-FFR means that future evidence may further refine the diagnostic paradigms discussed herein.
